# Marine *Pediococcus pentosaceus* E3 Probiotic Properties, Whole-Genome Sequence Analysis, and Safety Assessment

**DOI:** 10.1007/s12602-024-10283-7

**Published:** 2024-05-15

**Authors:** Eman H. Zaghloul, Nancy M. El Halfawy

**Affiliations:** 1 National Institute of Oceanography and Fisheries (NIOF), Cairo, Egypt; 2 Botany and Microbiology Department, Faculty of Science, Alexandria University, Alexandria, Egypt

**Keywords:** *Pediococcus pentosaceus*, Probiotic, Safety, Comparative genomics, Bacteriocin

## Abstract

Probiotics play a significant role in enhancing health, and they are well known for bacteriocins production. Evaluating probiotics’ whole-genome sequence provides insights into their consumption outcomes. Thus, genomic studies have a significant role in assessing the safety of probiotics more in-depth and offer valuable information regarding probiotics’ functional diversity, metabolic pathways, and health-promoting mechanisms. Marine *Pediococcus pentosaceus* E3, isolated from shrimp gut, exhibited beneficial properties, indicating its potential as a probiotic candidate. Phenotypically, E3 strain was susceptible to most antibiotics assessed, tolerant to low pH and high bile salt conditions, and revealed no hemolysin activity. Interestingly, E3-neutralized CFS revealed significant antibacterial activity against pathogens under investigation. Therefore, the concentrated CFS was prepared and evaluated as a natural biopreservative and showed outstanding antimicrobial activity. Furthermore, integrated-based genome assessment has provided insight into probiotic characteristics at the genomic level. Whole-genome sequencing analysis revealed that the E3 genome possesses 1805 protein-coding genes, and the genome size was about 1.8 Mb with a G + C content of 37.28%. Moreover, the genome revealed the absence of virulence factors and clinically related antibiotic genes. Moreover, several genes consistent with probiotic microorganisms’ features were estimated in the genome, including stress response, carbohydrate metabolism, and vitamin biosynthesis. In addition, several genes associated with survival and colonization within the gastrointestinal tract were also detected across the E3 genome. Therefore, the findings suggest that insights into the genetic characteristics of E3 guarantee the safety of the strain and facilitate future development of E3 isolate as a health-promoting probiotic and source of biopreservative.

## Introduction

Probiotics are living organisms with beneficial health effects when administered in adequate amounts, as described by the Food and Agriculture Organization of the United Nations (FAO) and the World Health Organization (WHO) [1]. Hence, to obtain the claimed health benefits, probiotic strains must be delivered to the host gastrointestinal tract (GIT) in an active physiological status [2]. Colonization of probiotics in GIT boosts the immune system and enhances the intestinal barrier [3]. Moreover, probiotic strains should be tolerant to gastric acid and bile salts, able to adhere to the GIT lining, [4]. Notably, the majority of probiotic lactic acid bacterium (LAB) strains are able to synthesize proteinaceous antibacterial metabolites, known as bacteriocins, that exert bactericidal or bacteriostatic activity against genetically closely associated bacteria [5]. In addition, they can synthesize a variety of vitamins [6], exopolysaccharides (EPS) [7], and amino acids [8].

*Pediococcus pentosaceus* is a gram-positive LAB with facultative anaerobic and carbohydrate degradation features [9, 10]. It can survive in the intestinal environment and exert probiotic activity [10]. Previous studies have reported the probiotic potential of *Ped. pentosaceus*, including obesity treatment, anti-inflammation, anticancer, antioxidant, antifungal, and cholesterol-lowering activity [11–14]. Several strains of *Ped. pentosaceus* have been reported to produce the bacteriocin pediocin PA-1/AcH. They also possess operons encoding other bacteriocins belonging to class IIa (anti-listerial bacteriocins), such as peniocin-A, and class III (bacteriolysins) bacteriocins. Anti-listerial bacteriocins are significant due to their effective bactericidal activity against *Listeria monocytogenes*; they also have the potential to act as biopreservatives in the food industry due to their ability to hinder the growth of many foodborne pathogens, including *Clostridium botulinum*, *Clostridium perfringens*, and *Staphylococcus aureus* [15]. In addition, these ribosomally synthesized bacteriocins have withdrawn the attention for potential pharmaceutical applications [16].

Nowadays, investigating the genome of probiotic strains is one of the currently recommended techniques and has significantly contributed to understanding the biotechnological potential of novel LAB strains [17]. Furthermore, analysis of the probiotic genome for identifying virulence and resistance to clinically relevant antibiotic genes offers valuable insights into the potential risks associated with their consumption [18–20]. In addition, the genome sequencing approach provides insights into functional diversity, metabolic pathways, and health-promoting mechanisms of probiotics [21].

Therefore, this study evaluated the marine isolate *Ped. pentosaceus* E3 phenotypically for probiotic properties. In addition, whole-genome sequencing (WGS) and functional annotation of E3 were performed to give insights into this strain’s safety, technological, and probiotic potential. Moreover, its ability to produce promising biopreservative was investigated.

## Materials and Methods

### Bacterial Strain Isolation, Morphological, and Biochemical Characterization

The marine isolate E3 was isolated from the gut of marine shrimp samples collected from the Mediterranean Sea. The gut was dissected and mixed well in 10 mL sterile saline; then, 1 mL was added to 9 mL of sterile De Man Rogosa and Sharpe broth (MRS; Merck, Germany) and incubated under anaerobic conditions at 37 °C for 24 h for enrichment, followed by successive streaking on MRS agar plates under same conditions until pure colonies were obtained. The isolate E3 was examined for gram’s staining and catalase reaction. E3 was biochemically identified as *Pediococcus pentosaceus* using the VITEK 2 microbial identification system version 07.01 (BioMérieux, France; http://www.biomerieux.com). In addition, the morphology and dimensions of isolate E3 cells were examined using scanning electron microscopy (SEM; JSM-IT 200, JEOL, Japan). The pure bacterial culture was preserved at − 20 °C in MRS broth enriched with 50% (v/v) glycerol for further investigations [22].

### Safety Assessment

#### Blood Hemolysis Activity

The hemolytic activity of *Ped. pentosaceus* E3 was assessed by streaking it on 7% (v/v) blood agar plates, followed by incubation for 24 h at 37 °C. After the incubation period, the plates were examined for signs of blood hemolysis [23].

#### Antibiotic Susceptibility Testing (AST)

The antibiotic susceptibility of *Ped. pentosaceus* E3 was evaluated using the Kirby-Bauer disc diffusion method. Antibiotic discs (Oxoid, UK) were placed on agar plates that had been previously inoculated with freshly prepared E3 culture. After being incubated for 24 h at 37 °C, the plates were checked to determine the presence of distinct regions devoid of bacterial growth surrounding the discs [24].

### Evaluating Probiotic Properties

#### Resistance to Low pH

E3 isolate was cultivated under various pH values to assess its capacity to endure low pH levels, mimicking the stomach’s acidic environment, which is estimated to have a pH of approximately 3.0 and a retention duration of approximately 6 h. The test was conducted using the methodology described by [22]. Briefly, a 1% (v/v) overnight culture of E3 isolate was inoculated into 20 mL of sterile MRS broth with various pH values (2.0, 3.0, 4.0, and 6.5). The mixture was then incubated at 37 °C for 6 h. The culture’s absorbance was measured at regular hourly intervals using a spectrophotometer (Fisher Scientific, USA) at OD_600_.

#### Bile Salt Tolerance

The variability in the gut’s average concentration of bile salts is estimated to be around 0.3% (w/v), with an average retention time of 4 h. Therefore, the capacity of E3 strain to endure elevated levels of bile salts was examined, employing the methodology described by [22]. A 1% (v/v) overnight culture of E3 was inoculated into 20 mL of sterile MRS broth containing different amounts of bile salts (0%, 0.1%, and 0.3% w/v). The mixture was then incubated at 37 °C for 6 h. The culture’s growth was measured at regular hourly intervals at OD_600_.

#### Antimicrobial Activity Assessment

The antimicrobial activity of *Ped. pentosaceus* E3 was assessed using the agar well diffusion technique against various gram-negative bacterial pathogens (*Klebsiella pneumonia* ATCC 13883, *Escherichia coli* ATCC 8739, *Pseudomonas aeruginosa* ATCC 27853), gram-positive bacterial pathogens (*Listeria monocytogenes*, *Staphylococcus aureus* ATCC 25923, *Enterococcus faecalis* ATCC 29212, *Bacillus subtilis* ATCC 6633), and fungus *Candida albicans* ATCC 10231. Bacterial inoculums were adjusted to the concentration of 10^6^ CFU/mL and were spread in Mueller–Hinton agar (MHA; HiMedia, India). Subsequently, wells of 6-mm diameter were punched into MHA plates and filled with 100 μL sterile cell-free supernatant (CFS) of E3, which had been neutralized (pH 7.0). After incubation at 37 °C for 24 h, the antibacterial activity was assessed by measuring the diameter of the inhibition zone. This assay was performed in duplicate for each bacterial strain, and the data were expressed as mean ± standard deviation (SD) [23].

### Genotypic Characterization

#### Whole-Genome Sequencing, Assembly, and Annotation

Genomic DNA was isolated using the GeneJET Genomic DNA Purification Kit (Thermo Fisher Scientific, UK), following the manufacturer’s instructions. WGS of E3 isolate was performed by MicrobesNG in July 2023 (Birmingham, UK; http://microbesng.uk) using the lllumina NovaSeq 6000 platform (Illumina, USA) with paired end reads of 250 bp in length. Genome annotation was performed using Prokka software (version 1.11) [24], the BV-BRC server (version 3.30.19*a*; https://www.bv-brc.org/) [25], and the NCBI Prokaryotic Genome Annotation Pipeline (PGAP) [26]. Metabolic pathways were predicted using the Kyoto Encyclopedia of Genes and Genomes (KEGG; http://www.genome.jp/kegg) [27] (accessed on 31 August 2023). Clusters of Orthologous Groups (COG) functional categories were analyzed using egg-NOG Mapper (http://eggnog-mapper.embl.de) (accessed on 1 September 2023).

#### Phylogenetic Analysis

The phylogenetic analysis of the 16S rRNA gene sequence of isolate E3 was analyzed using the NCBI’s BLASTn tools, resulting in the confirmation of the identification of isolate E3 as it showed a 99.75% similarity with *Ped. pentosaceus* strain DSM 20336 (accession number NR_042058.1). The 16S rRNA gene sequence of isolate E3 was matched with closely related species using the ClustalW tool (version 2.1). Subsequently, a phylogenetic tree was constructed using the maximum likelihood technique through the website www.phylogeny.fr (accessed on 27 September 2023) [28].

#### Genomic Aspects Related to Safety

The Resistance Gene Identifier (RGI) tool from the Comprehensive Antibiotic Resistance Database (CARD) [29] was utilized to predict antibiotic resistance genes (ARG). This tool was accessed through the BV-BRC server (version 3.30.19*a*). The VFanalyzer platform (https://www.mgc.ac.cn) was utilized to examine the virulence factors present in the genome. This platform may be accessed through the Virulence Factor Database (VFDB). The identification of plasmids and prophage sequences was conducted using the PlasmidFinder software (version 2.1) and the PHAge Search Tool Enhanced Release web server (PHASTER; https://phaster.ca) [30], respectively. The ISfinder site (https://www-is.biotoul.fr) was utilized to predict insertion sequences (IS) and transposons using BLASTn (version 2.2.31 +) [31]. The pathogenicity of E3 genome sequence was predicted using the PathogenFinder online tool provided by the Center for Genomic Epidemiology (https://cge.cbs.dtu.dk) [32].

#### Prediction of Carbohydrate Active Enzymes (CAZymes)

CAZyme-associated genes were identified in the E3 genome using dbCAN2 online server (https://bcb.unl.edu/dbCAN2/index.php) using DIAMOND blast search in CAZy database (http://www.cazy.org) with *E* value < 1e − 102 (accessed on 29 September 2023) [33].

#### Bacteriocin Gene Identification

BAGEL4 web tool and BLASTp were used to detect and visualize gene clusters involved in antimicrobial peptides biosynthesis (http://bagel4.molgenrug.nl) [34].

#### Comparative Genome Analyses

Genome sequences of twelve *Ped. pentosaceus* strains, namely MR001 (CP047081), 252371_901 (JAVAJL010000001), GBRCKU (JAMBZH010000010), LA0061 (CP137627), SL4 (CP006854), JQI-7 (CP023655), ZZ61 (CP129525), SS1-3 (CP023008), GDIAS001 (CP046938), SL001 (CP039378), PP16CP (JALCZR010000004), and ST65ACC (JAJHSK010000001) were selected and downloaded from NCBI database. Average nucleotide identity (ANI) calculation was calculated between genomes using Species WS (https://jspecies.ribohost.com/jspeciesws/) [35]. Global genome alignment was performed to check the synteny among large blocks of genomic sequences using progressive MAUVE [36].

#### Preparation of Concentrated CFS as a Natural Biopreservative

The concentrated E3 CFS was prepared following the methodology outlined by [37]. In this experiment, E3 was introduced into a 10 mL volume of MRS broth. The bacterial culture was then incubated statically at 37 °C for 24 h. Following this first incubation period, 1 mL of the culture was transferred to a larger volume of 100 mL of MRS broth and incubated at 37 °C for 48 h. The acquired culture was subjected to centrifugation at 6000 rpm for 15 min at 4 °C. Subsequently, it was neutralized (pH 7.0), filtered sterilized using a 0.22 μm Millipore filter, and concentrated through lyophilization in a freeze dryer (Christ, Germany). Before utilization, the lyophilized CFS was reconstituted in 5 mL of sterile distilled water to prepare the concentrated CFS (cCFS). The antimicrobial activity of the prepared cCFS was reevaluated against indicator pathogens, as mentioned above. Ampicillin (10 mg/mL) served as a positive control, while sterile qH_2_O was used as a negative control.

#### Genome Accession Number

The whole-genome shotgun project was submitted to the National Biotechnology Information Center (NCBI) GenBank database with the accession JAVLVE000000000.

## Results

### Morphological and Biochemical Characterization

**Fig. 1:**
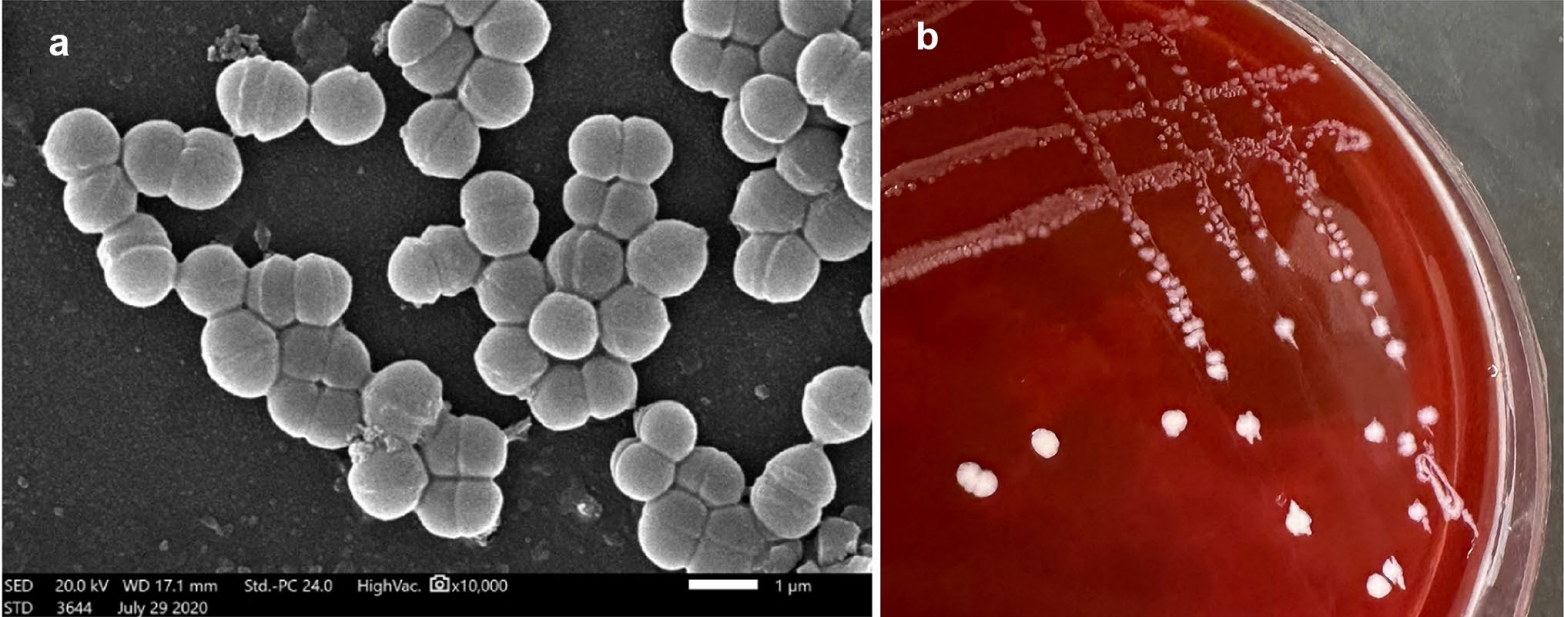
**a** Cell morphology of isolate E3 using a scanning electron microscope (SEM). **b** Growth of E3 on the surface of blood agar after 24 h of incubation at 37 °C

The bacterial isolate E3 was gram-positive and catalase negative. It revealed a coccoidal-shaped cell morphology (Fig. 1a). The isolate was biochemically identified using the VITEK 2 microbial identification system by 91% probability as *Ped. pentosaceus* (Table 1). E3 isolate was evaluated for hemolytic activity using blood agar plates, and the isolate was assigned as gamma-hemolytic bacteria as it lacked any signs of hemolytic activity (Fig. 1b).

**Table 1:** Biochemical characterization of E3 marine isolate using VITEK 2 system

**Test**	**Result**	**Test**	**Result**
D-AMYGDALIN (AMY)	** + **	D-GALACTOSE (dGAL)	** + **
PHOSPHATIDYLINOSITOL PHOSPHOLIPASE C (PIPLC)	**-**	D-RIBOSE (dRIB)	** + **
D-XYLOSE (dXYL)	**-**	L-LACTATE alkalinization (ILATK)	**-**
ARGININE DIHYDROLASE 1 (ADH1)	**-**	LACTOSE (LAC)	**-**
BETA-GALACTOSIDASE (BGAL)	**-**	N-ACETYL-D-GLUCOSAMINE (NAG)	** + **
ALPHA-GLUCOSIDASE (AGLU)	**-**	D-MALTOSE (dMAL)	** + **
Ala-Phe-Pro ARYLAMIDASE (APPA)	**-**	BACITRACIN RESISTANCE (BACI)	** + **
CYCLODEXTRIN (CDEX)	**-**	NOVOBIOCIN RESISTANCE (NOVO)	** + **
L-Aspartate ARYLAMIDASE (AspA)	**-**	GROWTH IN 6.5% NaCl (NC6.5)	**-**
BETA GALACTOPYRANOSIDASE (BGAR)	**-**	D-MANNITOL (dMAN)	**-**
ALPHA-MANNOSIDASE (AMAN)	**-**	D-MANNOSE (dMNE)	** + **
PHOSPHATASE (PHOS)	**-**	METHYL-B-D-GLUCOPYRANOSIDE (MBdG)	** + **
Leucine ARYLAMIDASE (LeuA)	**-**	PULLULAN (PUL)	**-**
L-Proline ARYLAMIDASE (ProA)	**-**	D-RAFFINOSE (dRAF)	**-**
BETA GLUCURONIDASE (BGURr)	**-**	O/129 RESISTANCE (comp. vibrio.) (O129R)	**-**
ALPHA-GALACTOSIDASE (AGAL)	**-**	SALICIN (SAL)	** + **
L-Pyrrolydonyl-ARYLAMIDASE (PyrA)	**-**	SACCHAROSE/SUCROSE (SAC)	**-**
BETA-GLUCURONIDASE (BGUR)	**-**	D-TREHALOSE (dTRE)	** + **
Alanine ARYLAMIDASE (AlaA)	** + **	ARGININE DIHYDROLASE 2 (ADH2s)	** + **
Tyrosine ARYLAMIDASE (TyrA)	**-**	OPTOCHIN RESISTANCE (OPTO)	** + **
D-SORBITOL (dSOR)	**-**	UREASE (URE)	**-**
POLYMIXIN B RESISTANCE (POLYB)	**-**		

### Antibiotic Susceptibility Testing

AST profile revealed that the isolate E3 was susceptible to the following antibiotics: vancomycin, azithromycin, linezolid, erythromycin, tetracycline, and ceftriaxone colistin sulfate. Moreover, the isolate exhibited resistance to teicoplanin, penicillin G, cefoxitin, sulphamethoxazol/trimethoprim, and colistin sulfate (Table 2).

**Table 2:** Antibiotic sensitivity testing of E3 isolate to selected antibiotics

**Antimicrobial**	Antibiotic resistance*
Teicoplanin (TEI, 30)	R
Vancomycin (VA, 30)	S
Azithromycin (AZM, 15)	S
Penicillin G (P, 1.5)	R
Cefoxitin (FOX, 30)	R
Linezolid (LZ, 30)	S
Erythromycin (E, 15)	S
Tetracycline (TE, 30)	S
Sulfamethoxazol/trimethoprim (SXT, 25)	R
Ceftriaxone (CTR, 30)	S
Colistin sulfate (CT, 10)	R

^*^Interpretation of the inhibition zone diameters is susceptible (S), intermediate (I), and resistant (R) according to CLSI (2017)

### Bile and Acid Tolerance

**Fig. 2:**
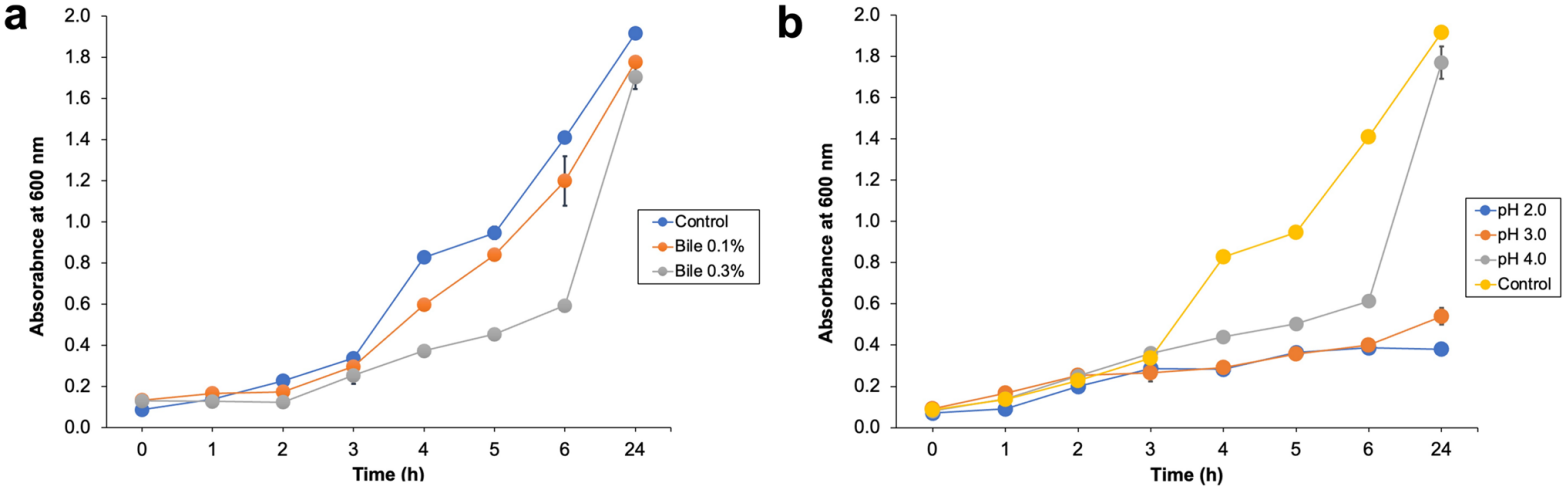
Effect of different **a** bile salt concentration and **b** pH values on growth of E3 at 37 °C for 24 h

E3 strain was assessed for bile and acid tolerance. It revealed survival potential in the presence of 0.1 and 0.3% bile salts over 24 h (Fig. 2a). Moreover, the effect of acidity on the viability of E3 strain was investigated. The isolate exhibited tolerance to low pH values (Fig. 2b). The results revealed that E3 isolate could survive in these conditions after 6 h of incubation.

### General Genomic Features

**Fig. 3:**
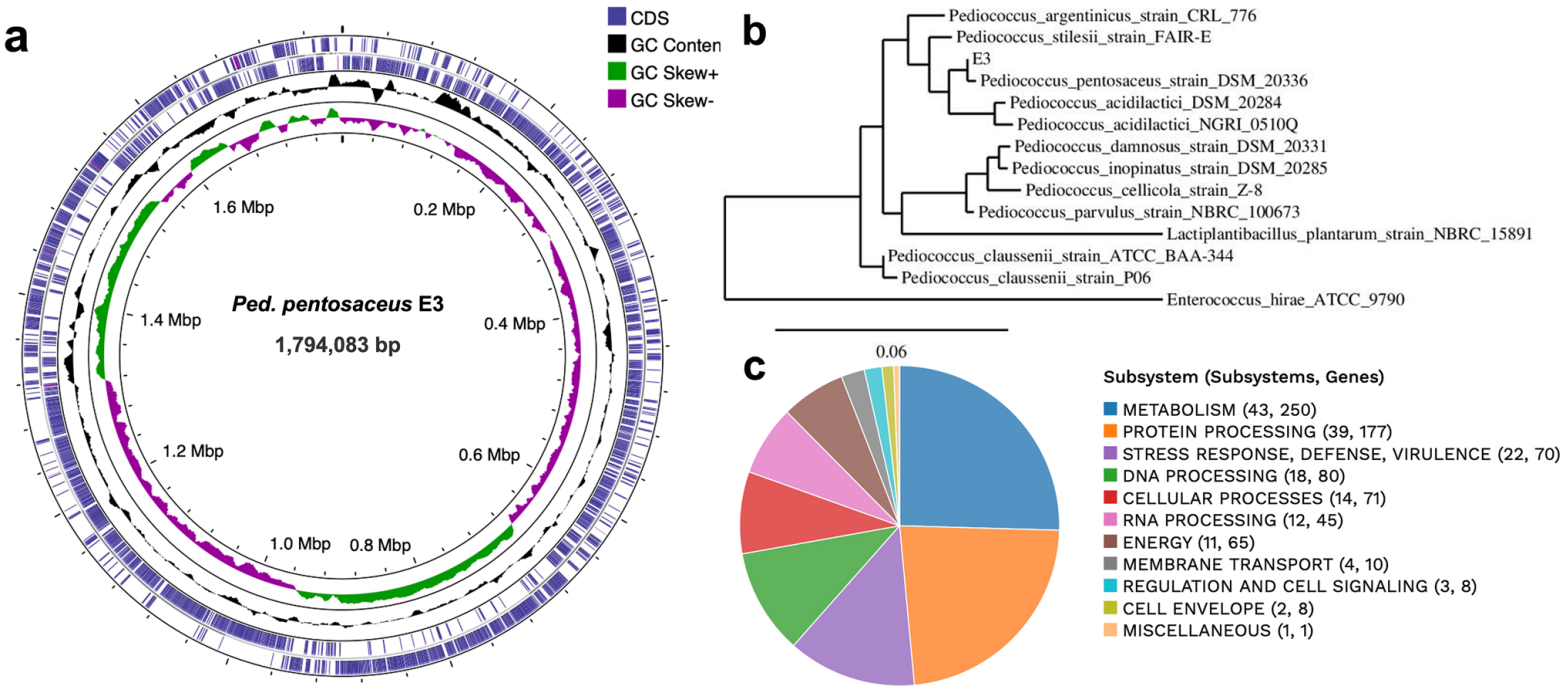
**a** Circular draft genome map of *Ped. pentosaceus* E3 generated using Proksee webserver (https://proksee.ca/). **b** Phylogenetic tree of the marine isolate E3 based on the 16S rRNA partial gene sequence. **c** Functional annotation overview of various subsystems available in E3 genome

E3 isolate whole-genome sequencing yielded 292,479 reads with a median insert size of 722 bases using Illumina NovaSeq 6000 with 30 × coverage. A total genome length of 1.8 Mb with a predicted 1805 protein-coding sequences (CDS) and a G + C content of 37.28% was estimated (Fig. 3a, Table 3). Using the BV-BRC server, genomic features, including 53 tRNA genes and 7 rRNA genes, were predicted in E3 genome sequence. Moreover, the phylogeny revealed that the E3 isolate was closely related and clustered with *Ped. pentosaceus* DSM20336 (Fig. 3b). KEGG pathways analysis revealed abundant functional categories of genes associated with carbohydrate and amino acid metabolism (Fig. 3c). Otherwise, the genome exhibited pathways related to lipids and xenobiotic metabolism. Additionally, isolate E3 was predicted as a non-human pathogen with a risk score of 0.078 and showed absence of pathogenic families.

**Table 3:** General genomic features of *Ped. pentosaceus* E3 genome assembly

**Assembly metrics**	***P. pentosaceus*** **E3**
Length (bp)	1,794,083
GC content (%)	37.28
CDS	1805
tRNA	53
rRNA	7
crispr array	1
Plasmids	0
Probability of being a human pathogen	0.078

### Assessment of E3 Genome Safety

The draft genome of *Ped. pentosaceus* E3 was mined for genetic determinants associated with the safety. The genome lacked genes encoding cytolysin (*cylA*), hyaluronidase (*hyl*), gelatinase (*gelE*), surface protein (*esp*), aggregation (*agg*), and adhesion collagen protein (*ace*), which is associated with virulence. Additionally, no clinically crucial genes were identified when investigating antibiotic-resistant genes (such as *vanA*, *vanB, vanC*). Furthermore, the occurrence of mobile genetic elements (MGEs) within the genome was investigated, and the results revealed the absence of insertion sequences (IS) associated with clinically relevant strains. Moreover, no plasmid replicons were predicted in the E3 genome sequence. One intact (PHAGE_Lactoc_bIL309) and two questionable (PHAGE_Lactob_iLp1308; PHAGE_Staphy_SPbeta_like) prophages were predicted in the E3 genome.

### Determination of Probiotic Characteristics

Genome annotation was screened for the presence of several probiotic-associated genes to verify the probiotic characteristics of the E3 strain at the genomic level. In silico analysis of the E3 genome revealed the presence of genes related to adhesion, stress resistance, temperature, and bile tolerance. *Ped. pentosaceus* E3 genome possessed the occurrence of several genes that facilitate cellular adhesion, including fibronectin-binding protein (*fbpA*), enolase (*eno*), and LPXTG-motif cell wall anchored protein (*srtA*). Moreover, bile tolerance encoded gene, cholylglycine hydrolase (*cbh*), and sodium proton antiport genes (*nhaC*, *nhaK*, *napA*) were predicted in the genome. Several genes encoding cold and heat stress were identified, including chaperones (*dnaK*, *dnaJ*, *hslO*), cold shock protein (*cspC*), and heat shock proteins (*grpE*, *groES*, *groEL*).

### Identification of Active Carbohydrate Enzymes

The E3 genome was found to contain genes involved in the carbohydrate’s metabolism and biosynthesis according to COG annotation. The CAZyme analysis revealed the presence of four families, including glycosyltransferase (GT), glycoside hydrolase (GH), carbohydrate-binding modules (CBM), and carbohydrate esterase (CE). The analysis showed 37 genes distributed in GT, 40 genes in the GH family, 4 in the CBM family, and 3 in the CE family.

### Determination of Antimicrobial Activity and Bacteriocin Identification

**Fig. 4:**
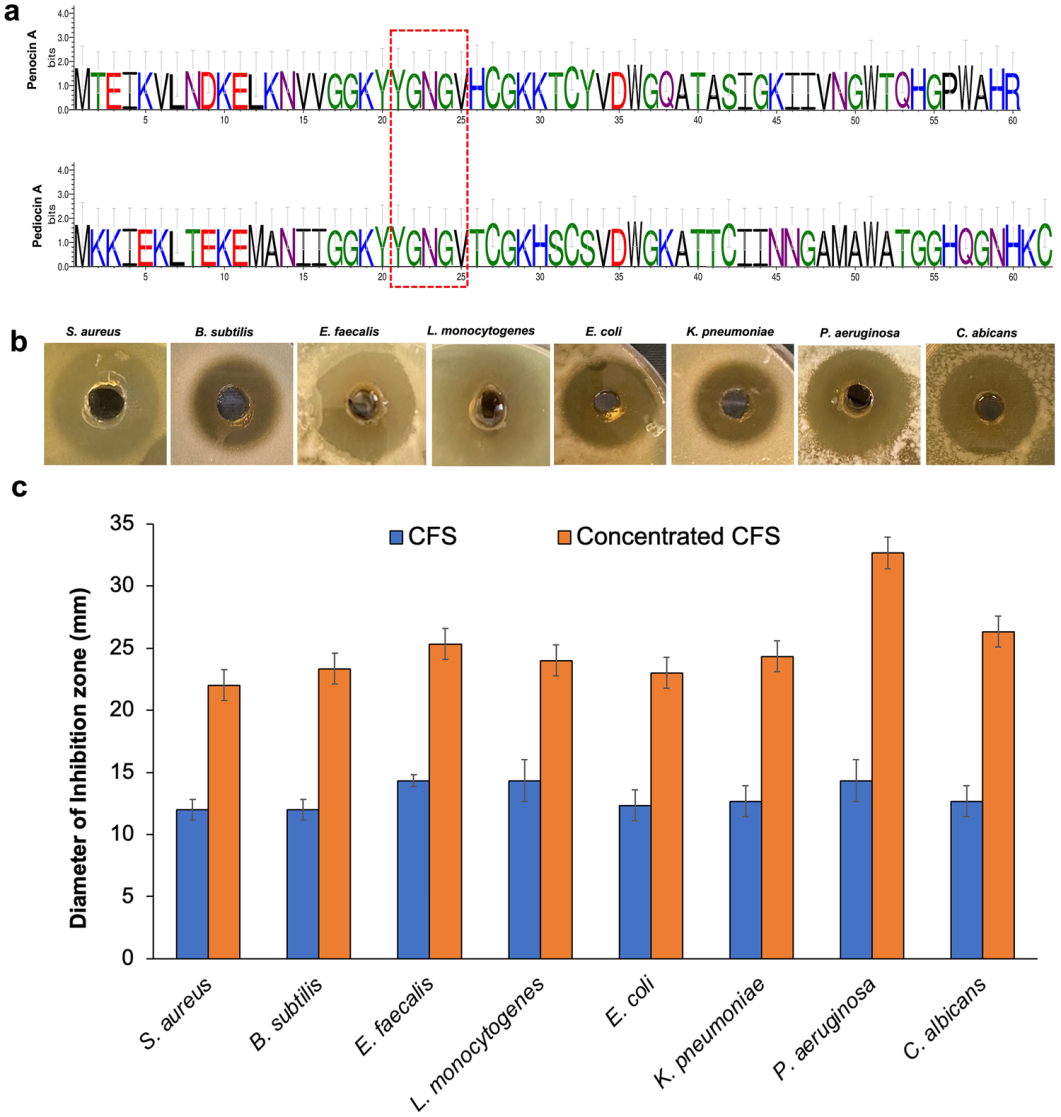
**a** Amino acid sequences of penocin A and pediocin A bacteriocins with highly conserved hydrophilic motif sequence (YGNGV) predicted with BAGEL4 web server and BLASTp in *Ped. pentosaceus* E3 genome. **b** Well diffusion method of cCFS. **c** Antimicrobial activity of neutralized CFS and cCFS against selected pathogens

Penocin A (60 aa) and Pediocin A (62 aa), class II anti-listerial bacteriocins, were predicted in the E3 genome. Structural genes encoding leader protein with a highly conserved hydrophilic motif included the motif YGNGV consensus, known as the “pediocin box.” Immunity protein enterocin A encoded with the *entA* gene was found adjacent to the bacteriocin structural genes (Fig. 4a). Moreover, neutralized CFS and cCFS revealed a potent antimicrobial activity against pathogens under investigation. Diameters of the inhibition zones ranged from 12.0 to 32.0 mm, and the mean results are shown with SD (Fig. 4b, c). In accordance with results obtained from the well diffusion assay, cCFS revealed significantly higher antibacterial activity than CFS. Furthermore, *P. aeruginosa* and *C. albicans* were the most affected microbial pathogens.

### Comparative Genome Analyses

**Fig. 5:**
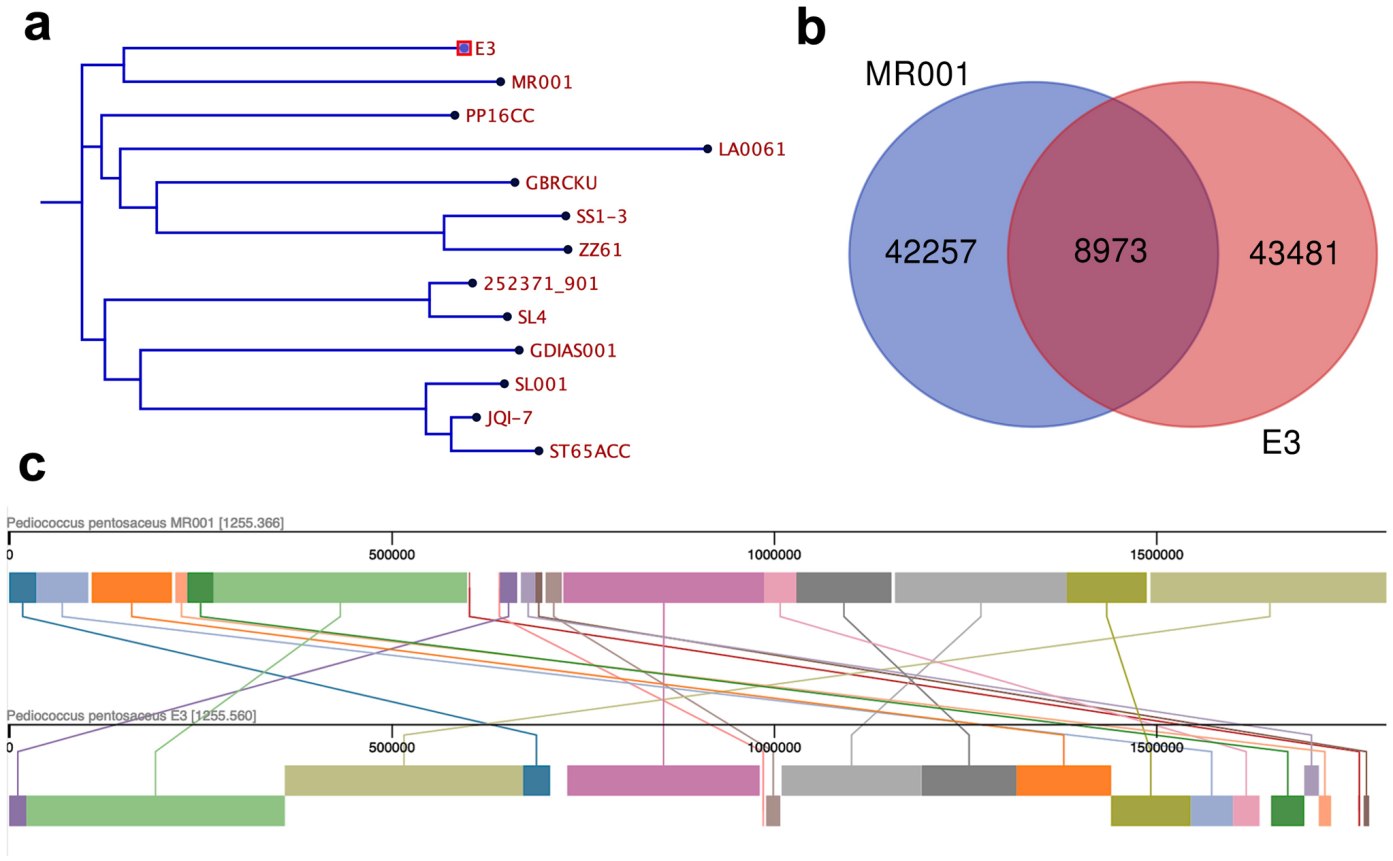
**a** Whole-genome-based phylogenetic tree of E3 isolate and 12 other *Ped. pentosaceus.* **b** Venn diagram showing the common genes in E3 and MR001 genome. **c** Global sequence alignment of the co-linear blocks using mauve alignment between E3 and MR001 genome

A comparative genome analysis was performed between E3 isolate and twelve other *Ped. pentosaceus* strains isolated from different sources. Average nucleotide identity (ANI) between E3 isolate and MR001 was the highest (98.84%). Notably, E3 isolate and *Ped. pentosaceus* MR001, a probiotic shrimp isolate (accession number CP047081), were closely related and clustered together in the whole-genome-based phylogenetic tree (Fig. 5a). Moreover, the two shrimp-derived strains showed 8973 shared genes (Fig. 5b). Mauve alignment revealed a certain similarity between isolate E3 and MR001 (Fig. 5c).

## Discussion

On a global scale, the rise of antimicrobial resistance (AMR) is a significant clinical health problem and is expected to result in 10 million deaths by 2050. AMR is widely recognized as a significant contributor to the proliferation of multidrug-resistant microbial strains. Conversely, the unintended harm to the gut microbiota resulting from antibiotics that target a wide range of bacteria contributes to the development of immune, metabolic, and neurological diseases in humans. Pursuing alternatives to conventional antibiotics is a highly sought-after goal [37].

Probiotics have become very popular recently due to their significance in producing therapeutics, natural antimicrobials, and food preservatives [38]. They have various characteristics that make them suitable alternatives to antibiotics [39]. Most probiotic strains already consumed in the food and aquaculture applications are not entirely studied at the genome level, resulting in the spread of virulence and ARG genes. To ensure the probiotic strains’ safety and effectiveness, it is crucial to analyze their entire genome sequences and confirm the absence of virulence, ARGs, and MGEs that are significant in clinical settings, providing vital knowledge about the potential hazards linked to their consumption [40]. Moreover, marine probiotics are adapted to endure harsh environmental circumstances, allowing them to develop distinctive traits and generate exclusive bioactive compounds with interesting biological properties and potential industrial uses, especially in the food and aquaculture fields [22].

Genomic analysis of novel marine probiotic strains is highly recommended as it greatly aids in recognizing the biotechnological capabilities of new marine LAB strains and offers valuable information regarding probiotics’ functional diversity, metabolic pathways, and health-promoting mechanisms. Therefore, in this study, genome-based analyses were performed to evaluate the safety and probiotic properties of marine *Ped. pentosaceus* E3 isolated from shrimp gut.

WGS of *Ped. pentosaceus* E3 strain revealed that the genome size and G + C content were similar to *Ped. pentosaceus* MR001, a probiotic shrimp isolate (accession number CP047081) [41]. Furthermore, the metabolic pathways in *Ped. pentosaceus* E3 strain were predicted through KEGG, and the analysis revealed the occurrence of various genes responsible for carbohydrates and amino acid metabolism that are considered critical probiotic features in LAB strains [42]. Moreover, the capacity of bacteria to utilize carbohydrates is an important indicator and provides the basis for further cultivation and selection of strains [43]. CAZyme analysis of the E3 genome revealed the presence of four family functions in carbohydrate metabolism. For instance, the GH family was the most abundant enzymes predicted in the E3 genome that play a significant role in carbohydrate glycosidic bond hydrolysis. In addition, GT2 and GT4 families, responsible for the synthesis of cellulose synthetase, chitin synthase, sucrose synthase, galactosyltransferase, and glucosyltransferase, were also predicted. These results agreed with CAZyme families detected in *Ped. pentosaceus* ST65AA genome, a promising probiotic candidate (accession number JAJHSK010000001) [42].

E3 isolate revealed no hemolytic activity, indicating this strain’s safety. Previous studies reported the absence of blood hemolysis in the probiotic *Pediococcus* spp. [44]. Furthermore, the E3 genome was screened for virulence, ARGs, and MGEs. Antimicrobial resistance is a major clinical health challenge as it may spread to other bacteria in the GIT. Genome screening of E3 isolate revealed no vancomycin resistance genes (*vanA*, *vanB*, *vanC*) and virulence traits. This suggests that marine *Ped. pentosaceus* E3 isolate could be safely used for food and feed. In addition, the stability of a probiotic strain is a significant factor in assessing its safety [45]. Notably, the E3 genome was devoid of plasmid replicons, which support the stability of the isolate against horizontal gene transfer (HGT).

The ability of marine probiotics to exert beneficial health effects on the host depends on the capacity to persist and colonize in the GIT [46]. Previous reports revealed that *Ped. pentosaceus* is an animal probiotic because it exhibits several health-promoting properties and can improve animal growth and the pathological state [47]. Probiotics are exposed to different stressful conditions when consumed by the host and pass through the GIT. First and foremost, they must tolerate the harsh conditions of the stomach. In agreement with the previous study, the E3 genome possesses genes involved in several types of stress responses, which support the probiotic properties of E3 isolate [42]. This discovery aligned with the results of the in vitro studies of *Ped. pentosaceus* E3, which demonstrated its capacity to endure acidic conditions with a pH of 3.0 and its ability to withstand a broad spectrum of bile salt concentrations. The ability to withstand the conditions of the small intestine and resist its environment may be more important than having a low tolerance for acidic pH. Research has indicated that strains of bacteria that are sensitive to acid can be buffered through the stomach. Nevertheless, in order for the bacteria to have a positive impact on the host, they need to possess the ability to withstand degradation caused by hydrolytic enzymes and bile salts in the intestine. Additionally, they need to survive and establish themselves in the small intestine. This characteristic is crucial when choosing new probiotic strains [48].

Bile salts harm living cells by modifying the cellular membrane structure. Probiotic strains can tolerate elevated levels of bile salts thanks to the existence of a distinct enzyme called bile salt hydrolase that aids in the breakdown of conjugated bile salts, reducing their toxicity. Alternatively, some dietary components can also mitigate the harmful impact of bile on microorganisms. Pre-exposure to acidic conditions for a duration of 3 h may induce the development of resistance to bile [22]. Similar results were documented by [49], who indicated that *Ped. pentosaceus* 1101 showed tolerance to acidity and bile salts. The genome of E3 revealed the presence of the cholylglycine hydrolase gene (*cbh*), which is a desirable property for selecting a probiotic strain. This finding agreed with *Ped. pentosaceus* MR001, which could tolerate different bile salt concentrations [41]. Furthermore, the heat shock chaperon DnaK and Na^+^/H^+^ antiporter, associated with acid adaptation, was predicted in the E3 genome. Additionally, the E3 genome analysis revealed the presence of cell wall anchored proteins (LPXTG) that function in the bacterial adhesion to epithelial cells of the GIT and contribute to its probiotic properties [50].

One of the key properties of probiotics is their ability to produce potent antimicrobial bacteriocins. These bacteriocins have proven efficacy against clinically significant pathogens such as *Streptococcus pneumoniae*, methicillin-resistant *Staphylococcus aureus* (MRSA), vancomycin-resistant Enterococci (VRE), and *Clostridium difficile*. Bacteriocins, classified as “generally recognized as safe” (GRAS, Grade One), are used as a safe food preservative due to their susceptibility to degradation by proteolytic enzymes in the mammalian GIT. Bacteriocins specifically target bacterial membranes and induce the rapid development of pores, even at extremely low concentrations ranging from picomolar to nanomolar [51].

Bacteriocins are often preferable to conventional antibiotics due to their limited range of action, allowing them to specifically eliminate targeted harmful bacteria without disrupting the beneficial commensal flora, which is a common drawback of antibiotics [51]. Previous studies have reported that probiotics’ antimicrobial production potential has a significant role in competing against GIT microbial pathogens [52]. The antimicrobial activity of E3 neutralized CFS against pathogens indicated its potential for bacteriocin production. Moreover, in silico, the detection of bacteriocin genes in the E3 genome revealed the presence of class II anti-listerial bacteriocins penocin A and pediocin A. This finding agreed with previously reported studies [42]. Pediocin PA-1 is an anti-listerial bacteriocin characterized by a highly conserved hydrophilic motif that includes the 5 amino acids (YGNGV), known as the pediocin box. Therefore, pediocin PA-1 exerts excellent potential as an antimicrobial agent against *Listeria monocytogenes*, a foodborne pathogen of particular concern in food industries [53]. Thus, bacteriocin producing E3 isolate could be used to compete with pathogenic bacteria for adhesion sites in intestinal epithelial cells [10]. Moreover, bacteriocins produced by E3 strain could be used as a bio-preservative in food owing to its promising antimicrobial activity.

Several studies suggested the therapeutic potential of probiotic *Ped. pentosaceus* strains. For instance, *Ped. pentosaceus* PP04 ameliorates high-fat diet-induced hyperlipidemia by regulating lipid metabolism in mice [54]. Furthermore, probiotic *Ped. pentosaceus* GS4 revealed anticancer potential against colon cancer cell lines [55]. In addition, *Ped. pentosaceus* AK-23 revealed an anti-inflammatory property in the host through regulating lipopolysaccharides (LPS) which is finally degraded to polysaccharides and fatty acids [47].

Overall, the in vitro and comprehensive genetic analyses of *Ped. pentosaceus* E3 genome revealed that the strain had no potential risk and confirmed the safety of this bacterium as a marine probiotic strain which facilitated its biotechnological potential in food industries.

## Conclusion

The current study emphasized the isolation and genomic analysis of the marine isolate identified as *Ped. pentosaceus* E3 with promising probiotic properties as it revealed no signs of blood hemolysis, ability to withstand low pH and high bile salt concentrations, and substantial antimicrobial activity. *Ped. pentosaceus* E3 genome analysis identified several significant genes related to stress adaption, adhesion, and the machinery responsible for the immunity and export of pediocin PA-1/AcH. The genomic assessments also demonstrated safety criteria, such as the lack of harmful characteristics and transmissible antibiotic resistance genes and plasmids. The findings from the WGS analysis suggest that *Ped. pentosaceus* E3 holds promise as a potential probiotic candidate with an ideal safety profile and indicate that it could have possible use in food bio-preservation.

## Data Availability

The article contains all the data generated during this study.

## Abbreviations

ARG: Antibiotic resistance genes
CDS: Coding sequence
COG: Clusters of Orthologous Groups
CFS: Cell-free supernatant
cCFS: Concentrated cell-free supernatant
EPS: Exopolysaccharide
FAO: Food and Agriculture Organization of the United Nations
GIT: Gastrointestinal tract
GO: Gene Ontology
HGT: Horizontal gene transfer
IS: Insertion sequence
KEGG: Kyoto Encyclopedia of Genes and Genomes
LAB: Lactic acid bacterium
MGEs: Mobile genetic elements
MRS: De Man–Rogosa–Sharpe
NCBI: National Center for Biotechnology Information
PGAP: Prokaryotic Genome Annotation Pipeline
SEM: Scanning electron microscopy
WGS: Whole-genome sequencing
